# Characterization and clinical verification of immune-related genes in hepatocellular carcinoma to aid prognosis evaluation and immunotherapy

**DOI:** 10.1186/s12885-023-10900-8

**Published:** 2023-06-15

**Authors:** Jialin Qu, Fenghao Sun, Yichen Hou, Haoran Qi, Xiaorong Sun, Ligang Xing

**Affiliations:** 1 Department of Radiation Oncology, Shandong Cancer Hospital and Institute, Shandong First Medical University, Shandong Academy of Medical Science, Jinan, 250117 Shandong China; 2 Department of Nuclear Medicine, Shandong Cancer Hospital and Institute, Shandong First Medical University, Shandong Academy of Medical Sciences, Jinan, 250117 Shandong China

**Keywords:** Immune-related genes-based prognostic index, Hepatocellular carcinoma, Tumor microenvironment, Prognosis, Immunotherapy

## Abstract

**Background:**

Immune-related genes (IRGs) have been confirmed to play an important role in tumorigenesis and tumor microenvironment formation in hepatocellular carcinoma (HCC). We investigated how IRGs regulates the HCC immunophenotype and thus affects the prognosis and response to immunotherapy.

**Methods:**

We investigated RNA expression of IRGs and developed an immune-related genes-based prognostic index (IRGPI) in HCC samples. Then, the influence of the IRGPI on the immune microenvironment was comprehensively analysed.

**Results:**

According to IRGPI, HCC patients are divided into two immune subtypes. A high IRGPI was characterized by an increased tumor mutation burden (TMB) and a poor prognosis. More CD8 + tumor infiltrating cells and expression of PD-L1 were observed in low IRGPI subtypes. Two immunotherapy cohorts confirmed patients with low IRGPI demonstrated significant therapeutic benefits. Multiplex immunofluorescence staining determined that there were more CD8 + T cells infiltrating into tumor microenvironment in IRGPI-low groups, and the survival time of these patients was longer.

**Conclusions:**

This study demonstrated that the IRGPI serve as a predictive prognostic biomarker and potential indicator for immunotherapy.

## Introduction

Hepatocellular carcinoma (HCC) is the most prevalent primary liver cancer and a leading cause of cancer-associated deaths across the globe. The incidence of hepatocellular carcinoma ranked sixth among all malignant tumors worldwide in 2020, although the mortality rate ranked third. [1]. Due to the gradual development of HCC, most patients have indeed missed the opportunity for surgery by the time they are diagnosed with HCC, resulting in a five-year survival rate of less than 30% across the globe. China, the country with the highest number of HCC cases, has a five-year survival rate of only 14.1% [2]. Early-stage patients who undergo surgical resection are often accompanied by a high recurrence rate [3]. Hence, developing new diagnostic and therapeutic strategies to predict and improve HCC prognosis is necessary.

Several breakthroughs have been made in the field of HCC treatment in recent years. Immunotherapy represented by immune checkpoint inhibitors (ICIs) has gained considerable attention in the treatment of HCC [4]. Meta-analyses have shown that ICIs can significantly improve overall survival (OS), progression-free survival (PFS), and overall response rate (ORR) compared with standard therapies [5–7]. The US Food and Drug Administration (FDA) has approved the targeted drug lenvatinib and the PD-1 blocker pembrolizumab for the first-line treatment of patients with advanced unresectable HCC that is not suitable for localized treatment [8]. However, only a small number of patients respond to ICIs. In HCC, the response rate to ICIs monotherapy is 15-23%, which increases to about 30% after combination therapy [9]. In addition, the price of immunotherapy is expensive, which may impose a heavy economicburden on some patients. Hence, there is a need to explore for identifying ideal cancer patients who may benefit from ICIs.

The biomarkers currently used, such as PD-L1 expression and tumor mutation burden (TMB), have their own limitations in predicting the efficacy of ICIs [10]. For example, patients with PD-L1 positivehave higher response rate to ICIs. However, some patients whose disease is PD-L1-negative by immunohistochemistry can still clinical benefit with anti-PD-1 or anti-PD-L1 therapies [11–13]. A clinical study showed that high TMB was not correlated with objective response to ICIs [14]. Moreover, the efficacy of ICIs depends not only on the features of tumor cells, but also on triggering the immune system to develop a long-lasting antitumor response [15]. Reported studies have revealed that the development of immune-related adverse events (irAEs) was associated with clinical benefits for HCC patients who were treated with ICIs [16]. Tumor-infiltrating lymphocytes (TILs) are an important component in the tumor microenvironment and that facilitates the anti-tumor immune response. The density of TIL in the tumor microenvironment has been verified to be closely related to the efficacy of ICIs [17]. The density of TIL in the tumor microenvironment has been verified to be closely related to the efficacy of ICIs [17]. In addition, the upregulation of immune-related genes such as T cell and NK cell proliferation genes indicates that ICIs have a more beneficial effect [18, 19]. These evidences suggest that immune-related genes may affect the efficacy of immunotherapy by regulating the number and activity of TILs. The immune microenvironment of HCC is extremely complex,[20]. so we aim to explore the relationship between immune related genes and immune microenvironment, prognosis and effect of immunotherapy in HCC. In this study, we used computational algorithms to analyze the gene-expression profiles of HCC and acquire an immune-related genes-based prognostic index (IRGPI). Besides, we classified the HCC into two subtypes as per the IRGPI. Conclusively, we established the IRGPI to characterize the clinical features and the various intra-tumoral immune landscape, which may precisely predict patient outcome and response to immunotherapy.

## Materials and methods

### Data collection and integration

We downloaded an RNA-seq transcriptome profiling dataset including 374 HCC and 50 normal samples, somatic structural variation and matching clinical information including age, sex, stage, tumor-node-metastasis classification over survival time and survival status of HCC from TCGA (https://portal.gdc.cancer.gov/). After removing invalid or partial data from the TCGA database, a total of 365 HCC patient transcriptome and clinical data were included in the training set for subsequent analysis (Table 1).

**Table 1:** Clinical characteritics of patients with HCC in TCGA database

Clinical information	Number	Percentage
**Total cases**	365	100
**Age**		
< 65	233	63.84
≥ 65	132	36.16
**Gender**		
Male	248	67.95
Female	117	32.05
**Grade**		
G1	55	15.07
G2	175	47.95
G3	121	33.15
G4	14	3.83
**Stage**		
Stage I	174	47.67
Stage II	86	23.56
Stage III	86	23.56
Stage IV	5	1.37
Unknow	14	3.84
**Tumor**		
T1	184	50.41
T2	90	24.66
T3	78	21.37
T4	13	3.56
**Node**		
N0	256	70.14
N1	4	1.09
Unknow	105	28.77
**Metastasis**		
M0	269	73.70
M1	5	1.37
Unknow	91	24.93

Moreover, 1,811 unique immune-related genes (IRGs) were obtained from the Immunology Database and Analysis Portal (ImmPort) database (https://www.immport.org/home) [21].

IMvigor210 dataset contains the transcriptome data and clinical information of 298 urothelial cancer patients who received PD-L1 antibodies. The dataset can be downloaded from http://research-pub.gene.com/IMvigor210CoreBiologies. In addition, we also collected information from 39 patients who used PD-1 antibodies from GSM1648114 (https://www.ncbi.nlm.nih.gov/geo/query/acc.cgi?acc=GSM1648114) and GSE18220 (https://www.ncbi.nlm.nih.gov/geo/query/acc.cgi?acc=GSE78220) datasets. We analyzed these patients to determine whether the model can predict the effectiveness of ICIs.

**Biological function analysis of differentially expressed immune-related genes (DEIRGs)**We retrieved immune-related gene expression data of HCC patients from the TCGA databases after gene name conversion and rectification of transcriptome data. DEIRGs between tumor tissues and normal tissues were identified by R package “limma” [22]. Heatmaps were drawn to visualize the differential expression of immune-related genes between tumor and normal tissues.

Next, we performed functional enrichment analysis on these genes to clarify the biological functions of DEIRGs, including Gene Ontology (GO) and Kyoto Encyclopedia of Genes and Genomes (KEGG). According to the criteria of false discovery rate (FDR) < 0.05, the top 10 most significant GO terms and KEGG signaling pathways were visualized by the R package “ggplot2” [23].

### Weighted gene co-expression network analysis (WGCNA)

We evaluated the 555 differentially expressed immune-related genes (DEIRGs) and constructed a gene co-expression network by R package “WGCNA”. [24]. Genes with a high level of topological overlap similarity would be integrated into a module in this network. Genes in the same module usually have a high degree of co-expression. In this study, we used two methods to identify the modular genes that have the most significant impact on clinical characteristics. The module eigengene (ME) represents the first principal component of the module, which is usually used to describe the expression pattern of the module. Module membership (MM) refers to the correlation coefficient between each gene in the same module, which is usually used to describe the reliability of a gene belonging to a module. Finally, we calculated the correlation between each module and the clinical characteristics to determine the module genes closely relevant to the clinical characteristics for subsequent analysis.

### Immune-related gene signature development and reliability evaluation

To explore IRGs highly related to overall survival (OS) and assess the prognostic evaluation, univariate Cox proportional hazard regression analysis was performed. With the cutoff value of *P* < 0.05, the prognosis-related IRGs were identified. of the optional IRGPI model based on prognosis-related IRGs was constructed using the Least Absolute Shrinkage and Selection Operator (LASSO) penalized Cox proportional hazards regression via R package ”glmnet” [25]. The IRGPI score of each HCC patient was calculated by the following formula:

$$IRGPIscore = \sum\limits_{i = 1}^n {Coefi*Xi}$$

(Herein, Coefi is the coefficient of each selected gene, while Xi is the expression value of IRGs.)

Each patient’s IRGPI score can be calculated using this formula. Patients with IRGPI score ≥ median value were classified as the IRGPI-high group, while those patients whose IRGPI score < median value were classified as the IRGPI-low group. Then, using the R package “survival”, a Kaplan-Meier analysis was used to compare the survival of IRGPI-high and IRGPI-low groups [26]. The IRGPI model’s independent prognostic relevance was further investigated using multivariate Cox regression.

### Analysis of gene mutation between IRGPI-high and low group

Based on the somatic mutation data downloaded from the TCGA database, we calculated the total number of non-synonymous mutations of each patient to obtain the TMB. The HCC driver genes were identified by the R package “maftool” [27]. We evaluated the frequency of driver gene mutations in the IRGPI-high and IRGPI-low groups, and the top 20 genes with the highest mutation frequency were designated as potential driver genes for HCC.

### Evaluation of tumor-infiltrating immune cells and immune function in the tumor microenvironment

The R package “CIBERSORT”, which is based on the principle of linear support vector regression, was used to calculate infiltration levels for different immune cells and immune function in HCC [28]. Immune and stromal contents for each HCC patient were evaluated by ESTIMATE [29]. The hierarchical agglomerative clustering of HCC was executed as per the tumor-infiltrating immune cells of each patient. Finally, a box plot was used to show the difference in immune infiltrating cells and immunological function between the IRGPI-high and IRGPI-low groups.

### Tumor Immune Dysfunction and Exclusion (TIDE)

TIDE is a computational framework to identify factors that underlie immune-dysfunction and immunosuppression of tumor immune escape. The TIDE score calculated by the computational framework consists of two parts: dysfunction score and exclusion score. The dysfunction and exclusion score can be calculated by multiplying the expression of immune-dysfunction and immunosuppression genes by their respective weight coefficients. Compared with the currently widely used biomarkers for evaluating the efficacy of immune checkpoint inhibitors (TMB, PD-L1 expression and IFN-γ), the TIDE score can better evaluate the efficacy of anti-PD-1 and anti-CTLA-4 treatments. To test the efficacy of ICIs, we calculated TIDE scores for patients in the IRGPI-high and low groups to examine whether there were any differences in TIDE scores between subgroups. The underlined study was conducted using the TIDE online application of ICIs response prediction, which is freely accessible with any modern web browser http://tide.dfci.harvard.edu/ [30].

### Multiplex immunofluorescence staining and quantitative analysis

30 surgical specimens of HCC from the Shandong Cancer Hospital and Institute and performed multiplex immunofluorescence staining. In order to ensure sufficient follow-up time and eliminate the shortened survival time caused by other reasons, all patients have good nutritional status, normal liver function and an estimated survival time of more than two years (Table 2). Tissue Sect. 4 μm thick were deparaffinized in xylene and then rehydrated in 100, 90, and 70% alcohol successively. Antigen retrieval was performed with boiling in antigen retrieval solution EDTA, endogenous peroxidase was inactivated by incubation in 3% H_2_O_2_ for 15 min. Next, the sections were pre-incubated with 10% normal goat serum and then incubated two hours or overnight with primary antibodies: MAPT (1:100 dilution, ab92676, Abcam), GHR (1:200 dilution, ab209790, Abcam), CD5L (1:200 dilution, ab45408, Abcam), CD8 (1:300 dilution, ab199016, Abcam), CCL14 (1:150 dilution, 14216-1-AP, proteintech). Subsequently, the sections were incubated with anti-mouse or anti-rabbit HRP-conjugated Polymer (Vector Lab, CA) for 10 min at room temperature. The antigenic binding sites were visualized using the OPAL dye. OPAL-520 (PerkinElmer Inc.), OPAL-690(PerkinElmer Inc.), OPAL-570(PerkinElmer Inc.), OPAL-780(PerkinElmer Inc.), OPAL-620(PerkinElmer Inc.) were applied to each antibody, respectively. After staining, all slides were counter-stained with DAPI for five minutes and mounted in Pro-Long Diamond Antifade Mountant (Thermo Fisher).

**Table 2:** Clinical characteristics of patients with HCC in hospital database

IRGPI group	IRGPI-low	IRGPI-high
**Total cases**	15	15
**Age**		
< 65	10	7
≥ 65	5	8
**Gender**		
Male	13	14
Female	1	2
**Grade**		
G1	6	3
G2	5	2
G3	4	7
G4	0	3
**Stage**		
Stage I	13	11
Stage II	2	4
**Tumor**		
T1	5	6
T2	10	9
**Node**		
N0	15	15
N1	0	0
**Metastasis**		
M0	15	15
M1	0	0
**Liver Function**		
Child-Pugh Score		
Child-Pugh A	15	15
Platelet Count		
(100–300) ×10^9^/L	15	15
Albumin		
(35–50) g/L	12	14
(28–35) g/L	3	1
Prothrombin Time		
11-13s	15	15
**Hepatitis-B infection**		
Yes	12	9
No	3	6

To obtain multispectral images, the stained slides were scanned using the Akoya CODEX system (Akoya Biosciences). Images were analyzed and quantified by Phenochart software (v.1.0.12, PerkinElmer Inc.). AI-assisted analyses using Phenochart software were performed to determine the recognition and levels of MAPT, GHR, CD5L, CCL14. In order to avoid bias caused by tumor heterogeneity, ten regions were selected randomly from each sample for fluorescence intensity analysis. The average fluorescence intensity of each protein in the ten regions was determined the expression level. Although bias cannot be completely eliminated, we are trying to reduce it. Individual cells were identified using the DAPI nucleus staining. CD8^+^ T cells were quantified and its percentages in each patient were calculated.

### Statistical analysis

All statistical analyses were carried out using R v 4.1.1 (www.r-project.org/), GraphPad Prism version 7.0 and SPSS version 21.0 software (IBM Corporation, Armonk, NY, USA). The Kruskal-Wallis test was used to compare differences between more than two groups, while the Wilcoxon test was used to compare two groups. The overall survival time of the two subgroups was evaluated by the Kaplan-Meier method and visualized by the survival curve. The statistically significant differences were evaluated by the log rank test. The correlation between IRGPI subtypes and clinical features was evaluated using the chi-square test, and the correlation coefficient was determined using the Spearman analysis. A p-value < 0.05 indicated statistical significance.

## Results

### Establishment of IRGPI in HCC

**Fig. 1:**
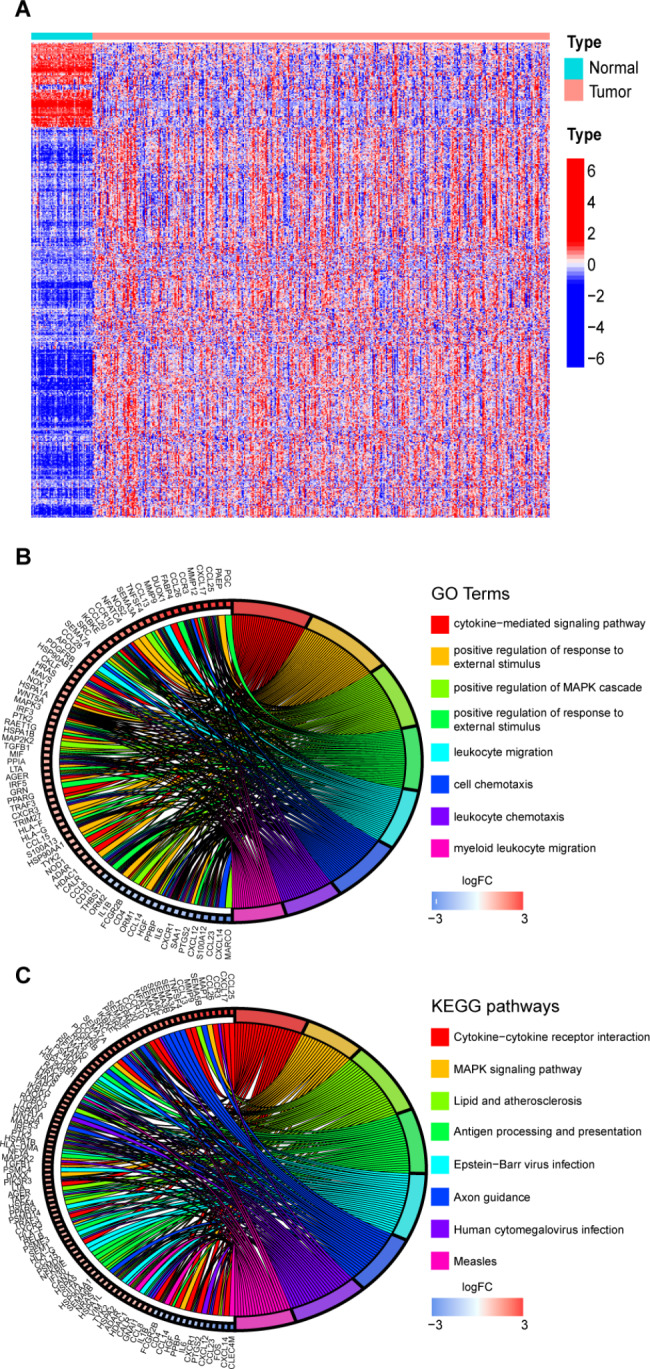
Different expression and function analysis of immune-related genes: **(A)** Different expression heatmap of 555 immune-related genes in normal and tumor tissues. **(B)** Go analysis indicated that these immune-related genes are mainly related to immune cell proliferation, cytokine response and immune cell migration. **(C)** KEGG analysis indicated that the signaling pathway includes cytokine receptor interaction, MAPK pathway and antigen processing and presentation

The TCGA database was used to obtain RNA-seq transcriptomic data, and the expression levels of immune-related genes in tumor and normal tissues were analyzed. The results showed that out of 1811 immune-related genes, 555 genes were found to be differentially expressed in tumor and normal tissues (*P* < 0.05) (Fig. 1A). These results suggested that immune-related genes play an indispensable role in the occurrence and development of HCC.

To further clarify the function of these genes, the underlined immune-related genes were selected as candidates for Gene Ontology (GO) and Kyoto Encyclopedia of Genes and Genomes (KEGG) enrichment analysis. GO analysis explains the roles of these genes using three aspects: cellular component (CC), molecular function (MF), and biological process (BP). The results of GO analysis suggest that the functions of these genes are mainly focused on the positive regulation of immunity such as cell proliferation, cytokine secretion and cell chemotaxis (Fig. 1B). KEGG analysis was performed to investigate the pathways involved in the regulation of downstream genes by immune-related genes. The anti-tumor immune response is closely associated with mitogen-activated protein kinase (MAPK) and antigen processing and presentation, which are the most critical pathways (Fig. 1C).

**Fig. 2:**
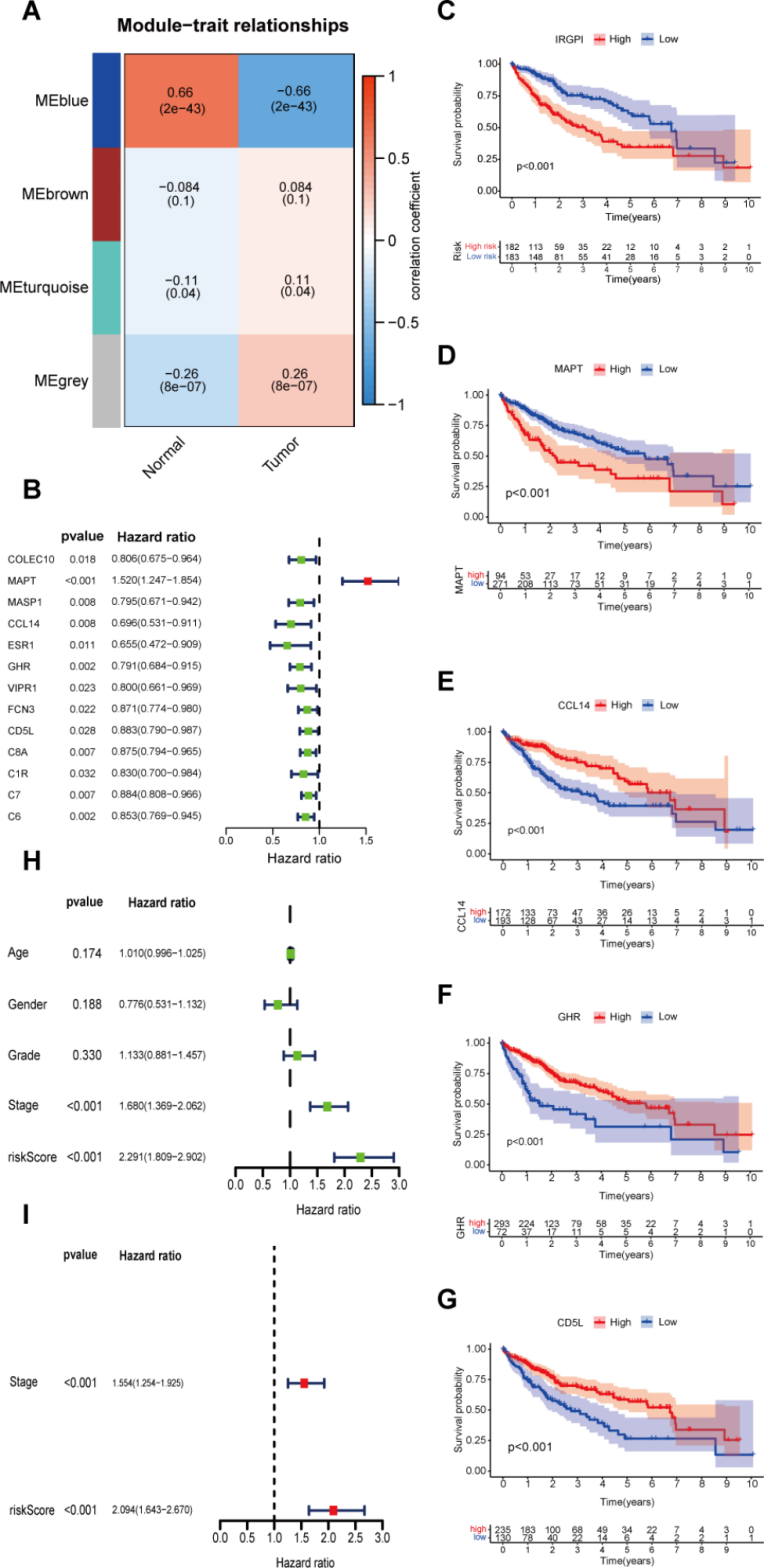
Construction of IRGPI: **(A)** Weighted Gene Co-expression Network Analysis (WGCNA) immune-related genes and their four modules based on their degree of co-expression. The number in the module indicates the correlation between the module genes, HCC, and the p-values. **(B)** Univariate Cox proportional hazard regression analysis screens out the genes that have the greatest impact on the survival time of HCC and hazard ratio. **(C)** K-M curves for high and low IRGPI groups in the TCGA cohort. Log rank test, p < 0.001. **(D)** K-M curves for high and low MAPT expression groups in the TCGA cohort. Log rank test, p < 0.001. **(E)** K-M curves for high and low CCL14 expression groups in the TCGA cohort. Log rank test, p < 0.001. **(F)** K-M curves for high and low GHR expression groups in the TCGA cohort. Log rank test, p < 0.001. **(G)** K-M curves for high and low CD5L expression groups in the TCGA cohort. Log rank test, p < 0.001. **(H, I)** Univariate and multivariate independent prognostic analyses of IRGPI.

WGCNA was performed to identify the co-expressed gene modules among the 555 DEIRGs, and to investigate the association between genes and HCC. The results obtained from WGCNA show that the genes in the blue module are closely related to the occurrence of HCC (R = 0.66, p-value = 2e-43) (Fig. 2A). Therefore, 58 genes from the blue module were selected for further study.

Univariate Cox proportional hazard regression analysis identified 13 genes that are closely associated with the prognosis of HCC (p-value < 0.05, Fig. 2B). LASSO regression prevents overfitting through variable selection and regularization, which helps to improve the accuracy of the model. After minimizing overfitting by LASSO regression, 4 genes were selected as hub IRGs of the model: MAPT, CCL14, GHR and CD5L. Therefore, IRGPI was derived by multiplying hub IRGs with the univariate COX regression coefficient as follows: IRGPI score = [expression of MAPT × 0.401232]. + [expression of CCL14 × -0.22304]. + [expression of GHR × -0.19147]. + [expression of CD5L × -0.09349].

### IRGPI predicts survival for HCC patients

According to the median IRGPI score, 374 HCC patients from the TCGA database were classified into IRGPI-low and IRGPI-high subgroups. Finally, a survival-prognosis model was established, using Kaplan-Meier (K-M) method. The K-M survival analysis of the model showed that the survival period of patients in the IRGPI-low group was significantly longer than that of the IRGPI-high group, which implied a remarkable ability in differentiating good or poor clinical outcomes among the two subgroups (Fig. 2C). Moreover, based on the 4 hub genes in the model, we divided patients into high and low-expression groups, followed by performing survival analysis. The results showed that patients with high expression of MAPT had a poor prognosis, while patients with high expression of the other three genes had a longer survival time. Consistent with the results of the above Univariate Cox regression analysis, the obtained results reveal that MAPT is a high-risk gene while the remaining genes are low-risk genes (Fig. 2D-G).

We further used univariate and multivariate independent prognostic analyses to evaluate the predictive value of the prognostic model. All independent prognostic analysis results were consistent, which shows that the IRGPI can be independent of other clinical characteristics as an independent prognostic factor (Fig. 2H, I).

### IRGPI significantly related to the disease progression, TMB and driver gene mutations

**Fig. 3:**
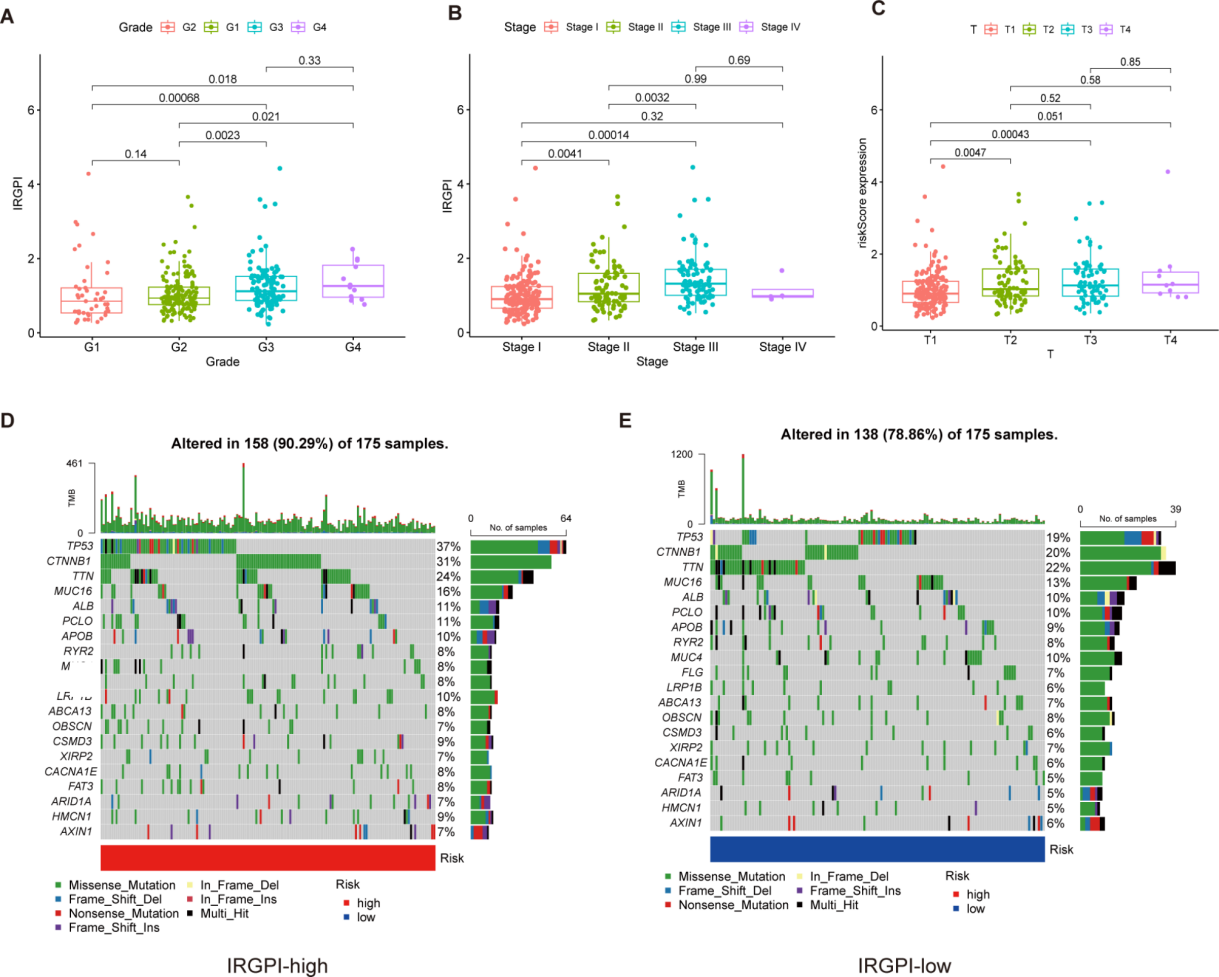
Clinical features and drive gene mutation in IRGPI-low and IRGPI-high groups: **(A-C)** The relationship of IRGPI and clinical characteristics in HCC. **(D)** Twenty genes, mutation types and TMB with the highest mutation frequency in the IRGPI-high group. **(E)** Twenty genes, mutation types and TMB with the highest mutation frequency in the IRGPI-low group

The correlation analysis was performed via a chi-square test to explore the possible correlation between IRGPI and clinicopathologic factors. The results showed that patients in the IRGPI-high group have higher tumor pathological grades, which implied a higher degree of tumor malignancy (Fig. 3A). Furthermore, patients in the IRGPI-high group have higher clinical stages and a higher tumor infiltration area (Fig. 3B, C). This suggests that IRGPI-high patients had more aggressive tumors, faster tumorigenesis, and a poorer prognosis.

Herein, this study identified the 20 most often mutated genes as driver genes for HCC by studying the somatic structural variation of HCC. Moreover, the frequency of driver gene mutations in the IRGPI-high group was found to be significantly higher than that in the IRGPI-low group, with a higher TMB (Fig. 3D, E).

### IRGPI predicts tumor infiltrating immune cells and immune function in the microenvironment

**Fig. 4:**
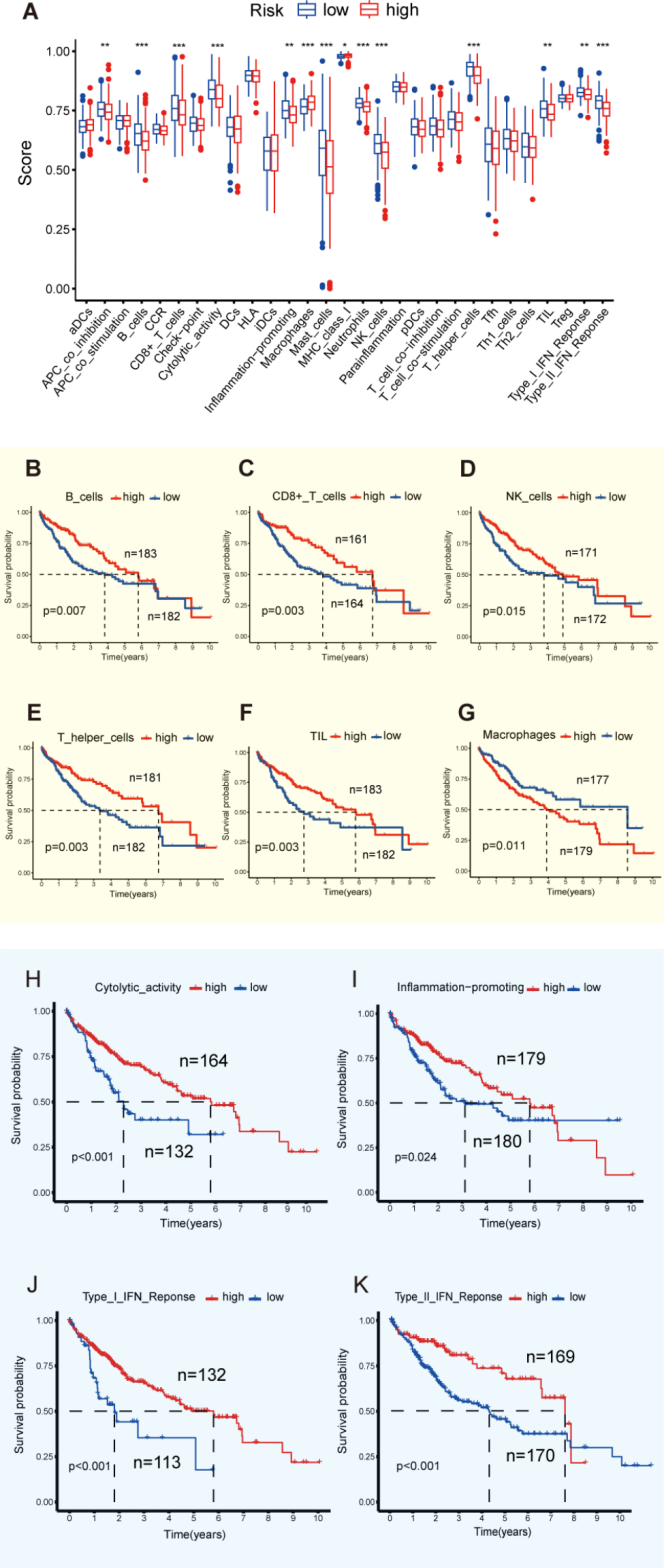
The landscape of immune cell infiltration in the tumor microenvironment of HCC: **(A)** Differences in immune infiltrating cells and immune function in tumor microenvironment between IRGPI-high and low group. *, p < 0.05; **, p < 0.01; ***, p < 0.001. **(B-G)** K-M curves for high and low immune infiltrating cells in the TCGA cohort. **(H-I)** K-M curves for high and low immune function in the TCGA cohort

Immune infiltrating cells is one of the important factors affecting immunotherapy. It is necessary to study the abundance and function of immune infiltrating cells between the two groups [17]. CIBERSORT was used to evaluate the relative proportion of 29 types of immune cells and immune function influencing the procedure of anti-tumor immune response in HCC. It is a useful tool for analyzing immune infiltrating cells in the tumor microenvironment. The results showed that the number of tumor-infiltrating cells in the IRGPI-low group was significantly greater than that of the IRGPI-high. Among the 14 immune infiltrating cells, patients in the IRGPI-low group have a higher abundance of B cells, CD^8+^ T cells, mast cells, neutrophils, NK cells, and T helper cells, but the abundance of macrophages is lower. In terms of immune function, patients in the IRGPI-low group are more active than IRGPI-high group, such as cytolytic activity, inflammation-promoting, and response to interferon (IFN) (Fig. 4A).

Herein, the survival of patients was analyzed based on immune cells. According to the obtained results, the effector cells in anti-tumor immune response such as B cells, CD8^+^ T cells, NK cells, T helper cells and TILs were associated with a good prognosis. Furthermore, macrophages that can secrete immune negative regulatory factors to promote tumor cell immune escape predicted poor survival. (Fig. 4B-G) The underlined results are also supported by preliminary research [31–34]. Furthermore, the impact of immune function on the prognosis was analyzed which revealed that the pro-inflammatory factors that promote the activity of immune cells in the tumor microenvironment are related to a good prognosis (Fig. 4H-K).

**Fig. 5:**
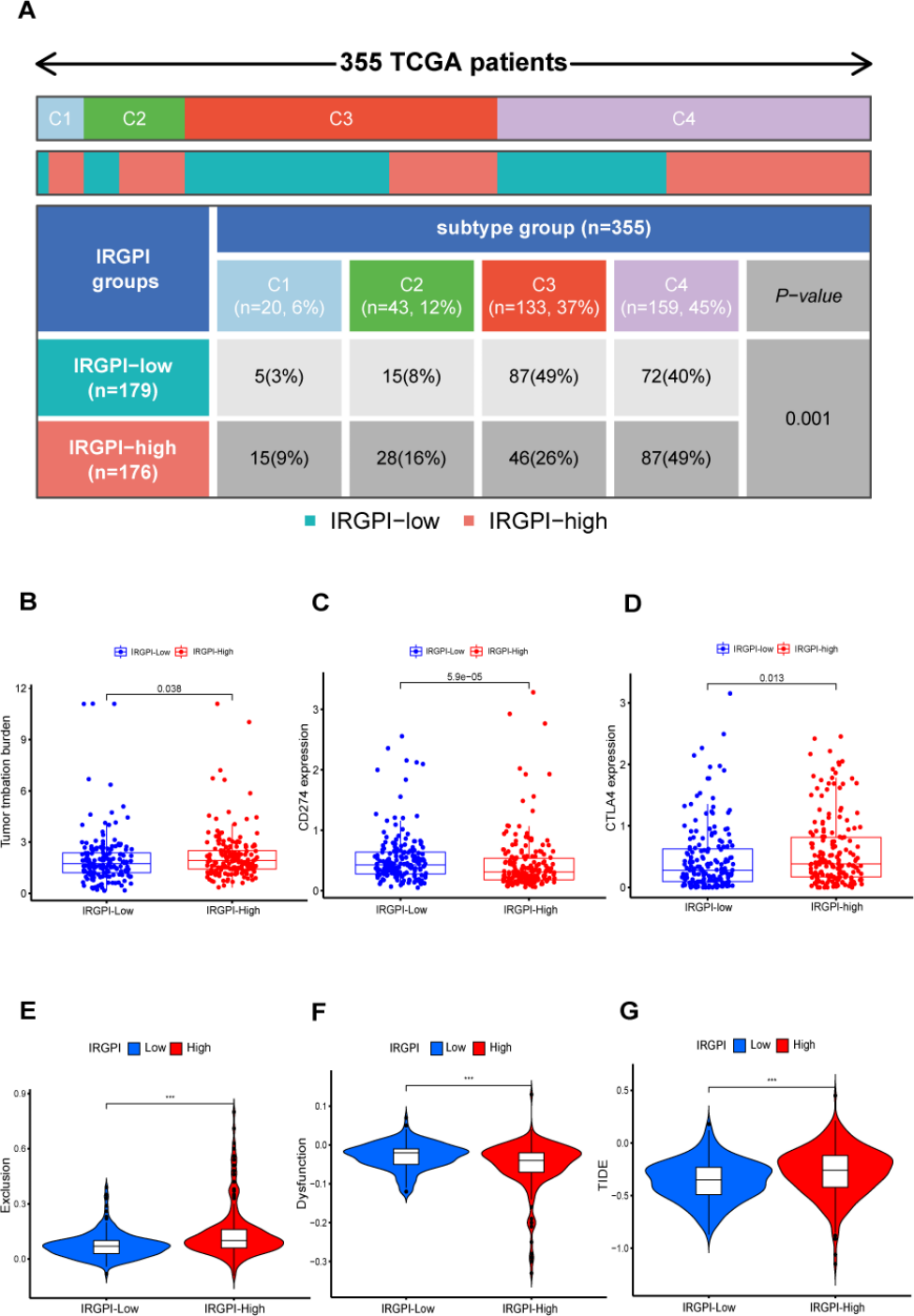
The correlation between the IRGPI and immune subtype, immune checkpoint and immune escape: **(A)** The distribution of patients in IRGPI-high and low groups in different TCGA immunotypes. C1:wound healing; C2: IFN-γ dominant; C3: inflammatory; C4: lymphocyte depleted. chi-square test, p < 0.001. **(B)** TMB difference in the IRGPI-high and low subgroups. Wilcoxon test, p = 0.038. **(C)** PD-L1 (CD274) expression difference in the IRGPI-high and low subgroups. Wilcoxon test, p < 0.001. **(D)** CTLA-4 expression difference in the IRGPI-high and low subgroups. Wilcoxon test, p = 0.013. **(E)** Immune exclusion score in the IRGPI-high and low subgroups. Wilcoxon test, p < 0.001. **(F)** Immune dysfunction score in the IRGPI-high and low subgroups. Wilcoxon test, p < 0.001. **(G)** TIDE score in the IRGPI-high and low subgroups. Wilcoxon test, p < 0.001

The immunogenicity of more than 10,000 tumor samples of 33 cancers from TCGA has already been reported, calculating the correlation coefficients among 160 immune characteristics. Furthermore, cluster analysis was performed to obtain the immune expression characteristics of 5 core modules. According to these 5 immune expression characteristics, all non-hematological tumors in the TCGA database are clustered into 6 immune subtypes: wound healing, IFN-γ dominant, inflammatory, lymphocyte depleted, immunologically quiet, and TGF-β dominant (C1-C6). The survival analysis of 6 immune subtypes revealed that C3 had the best prognosis, followed by C1 and C2, and C4 and C6 had the worst prognosis [35]. Matching the IRGPI group with the TCGA immune subtypes, we found that half of the patients in the low-IRGPI group belonged to the C3 with the best prognosis. Three-quarters of patients in the high-IRGPI group are C1, C2, or C4 with relatively poor prognoses (Fig. 5A).

### IRGPI predicts responses of immunotherapy

To verify whether the score can accurately predict the patient’s response to ICIs, herein, the difference between traditional biomarkers and the TIDE score between the IRGPI-low group and the high were analyzed. The results showed that the TMB of the IRGPI-high group is greater than the IRGPI-low group. (Fig. 5B) Regarding the expression of immune checkpoint molecules, the expression of programmed cell death-ligand 1 (PD-L1, CD274) was considerably higher in the IRGPI-low group than in the IRGPI-high group. However, the expression of cytotoxic T-lymphocyte-associated protein 4 (CTLA-4) was the opposite (Fig. 5C, D).

Recent studies have revealed that tumors have two different immune escape mechanisms. Even when a substantial number of cytotoxic T lymphocytes infiltrate the microenvironment of certain cancers, their functions are vague under the influence of immunosuppressive molecules [36]. In some tumors, immune-negative regulatory cells and factors can eliminate T cells infiltrating the tumor tissues and form a state of immune exclusion [37]. Therefore, the researchers have designed a new computing architecture: the TIDE score. It is also believed that TIDE score values can replace a single biomarker predicting the efficacy of ICIs [30]. Herein, TIDE scores were performed on all patients which revealed that patients in the IRGPI-high group had higher immune exclusion levels. (Fig. 5E) The finding indicated that fewer immune cells were infiltrating the tumor microenvironment, which was consistent with the outcomes of our analysis of immune cells. However, patients in the IRGPI-low group are more in an immune dysfunction state. (Fig. 5F) Previous studies have shown that patients with immune exclusion are resistant to ICIs, and the treatment effect is not as good as that of immune dysfunction [38]. Furthermore, the TIDE score of the IRGPI-high group was significantly higher than the IRGPI-low group, which means that the immunotherapy effect of the high-risk group is poor (Fig. 5G).

**Fig. 6:**
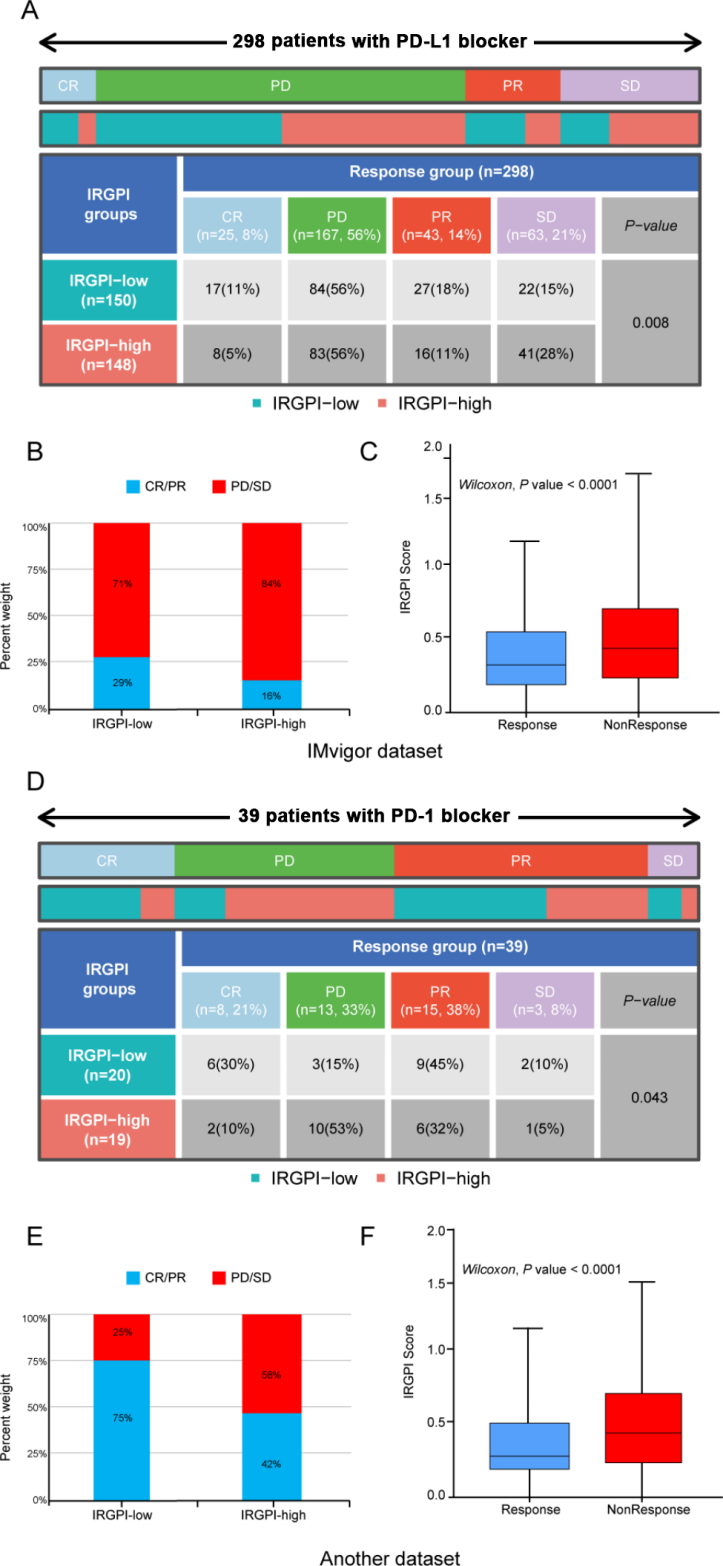
The role of IRGPI in the prediction of ICIs benefits: **(A)** The response of IRGPI-high and IRGPI-low urothelial carcinoma patients to PD-L1 inhibitors. chi-square test, p = 0.008. **(B)** The proportion of patients in IRGPI-high and low groups who responded to PD-L1 inhibitors. **(C)** IRGPI of patients in the PD-L1 inhibitor response and non-response group. Wilcoxon test, p < 0.0001. **(D)** The response of patients with melanoma and NSCLC in the IRGPI-high and low group to PD-1 inhibitors. chi-square test, p = 0.043. **(E)** The proportion of patients in IRGPI-high and low groups who responded to PD-1 inhibitors. **(F)** IRGPI of patients in the PD-1 inhibitor response and non-response group. Wilcoxon test, p < 0.0001

To further validate the accuracy of the model’s ability to predict the effect of immunotherapy, we analyzed three immunotherapy datasets. The IMvigor210 cohort contains 298 patients with urothelial cancer treated with PD-L1 blockers. Each patient was scored and assigned to IRGPI-high and low groups according to the median value. Patients with complete response (CR) and partial response (PR) were defined as a response, and patients with stable disease (SD) and progressive disease (PD) were defined as a non-response. Notably, the objective response rate (ORR) of the PD-L1 blocker was higher in the IRGPI-low than in the IRGPI-high group in the IMvigor210 cohort (chi-square test, P = 0.008), and the IRGPI of non-responders were significantly higher than responders (Wilcoxon, p-value < 0.001) (Fig. 6A-C). A similar outcome was observed in the GSE78220 and GSE67501 cohort, which contains 39 patients with melanoma and non-small cell lung cancer (NSCLC) treated with PD-1 therapy (chi-square test, P = 0.043) (Fig. 6D-F). Taken together, the IRGPI can effectively predict the response to ICIs.

### Clinical verification

**Fig. 7:**
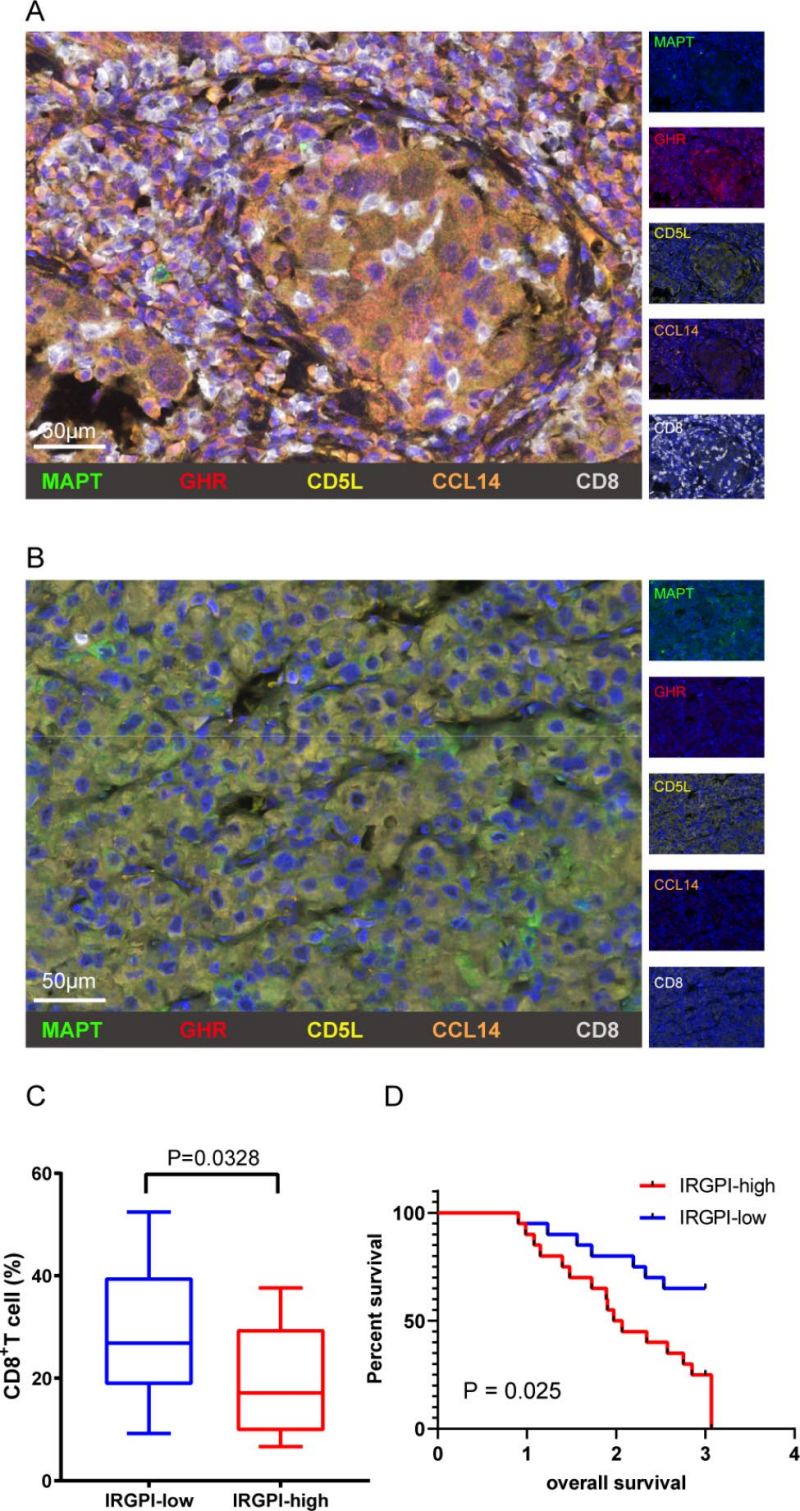
Two different tumor microenvironment of CD8^+^T cells infiltrated in IRGPI-high and IRGPI-low: **(A, B)** Representative multiplex immunofluorescence images to show the distribution of CD8 + T cells in IRGPI-low and IRGPI-high, respectively: MAPT (green), GHR (red), CD5L (yellow), CCL14 (orange), CD8 (white) and DAPI (blue). Scale bar, 50 μm. **(C)** Statistical graphs to show the proportion of CD8 + T cells between IRGPI-low and IRGPI-high groups. p = 0.0328; Wilcoxon test. **(D)** K-M curves for IRGPI-high and low in the hospital cohort. p = 0.025

Immunostaining on the HCC cohort further confirmed that CD8^+^ T cells were more abundant in IRGPI-low group, while the tumor microenvironment of IRGPI-high group presents a scene of immune desert (Fig. 7A, B). Statistical analysis of the number of CD8^+^ T cells showed that the proportion of CD8^+^ T cells in the IRGPI-low group was significantly increased than that in the IRGPI-high group (Fig. 7C). The results of survival analysis showed that the survival time of IRGPI-low group was longer that IRGPI-high group (Fig. 7D). Together, these results indicate that IRGPI-low group has a unique immune ecosystem, with increased CD8^+^ T cells. This is one reason why patients in the IRGPI-low group responded better to ICIs.

## Discussion

With major advances in immunotherapy for progressive solid tumors in recent years, clinical investigators have conducted numerous explorations in HCC. Ranging from KEYNOTE-524 and IMbrave-150, the indications of immunotherapy in HCC have been broadened from second/third-line treatment to first-line [8, 39]. Consequently, the rapid development of immunotherapy will potentially transform HCC into a chronic condition with a long-life expectancy. However, a significant limitation of immunotherapy in HCC is that only a minority of patients have benefitted from it. Even the Society for Immunotherapy of Cancer (SITC), who published the first clinical practice guideline on immunotherapy for the treatment of HCC, has emphasized that suitable patients for immunotherapy should be identified [40]. Immune-related genes have been confirmed to play an important role in tumorigenesis and tumor microenvironment formation [41]. For HCC, the influence of immune-related genes on prognosis and response to immunotherapy is worth exploring. In this study, we used a bioinformatic methodology to establish a prognostic model: IRGPI. The outcome of our subsequent analysis revealed that the IRGPI is a potential biomarker in assessing the prognosis and response to immunotherapy in HCC.

As compared to other tissues, liver tissue expresses GHR abundantly. In the liver, GHR downregulation may interfere with the GH signaling pathway. Study found that the expression of GHR and CDKN1A, one of the key inhibitors of cell cycle, were significantly down-regulated in hepatocellular carcinoma [42]. Furthermore, GH-mediated STAT5 prevent cell proliferation by activating CDKN1A and CDKN2B transcription [43]. CD5L, also know as apoptosis inhibitor of macrophages (AIM), has been observed to be down-regulated in HCC and associated with poor prognosis [44]. CD5L can promote the cell death of HCC through complement activation [45]. CCL14 inhibits the proliferation and promotes apoptosis of HCC by modulating the activation of Wnt / β-catenin pathway [46]. Microtubule-associated protein tau (MAPT), play a key role in tubulin assembly and microtubule stabilization,[47]. MAPT is overexpressed in a variety of tumor cells, including HCC, which promotes tumor cell proliferation and metastasis and induces tumor cell resistance to paclitaxel [48–50].

According to the IRGPI, HCC patients was categorized into distinct subtypes. Compared with the IRGPI-high group, the IRGPI-low group has lower pathological grade, clinical stage and longer survival time. The frequency of driver gene mutations such as TP53, CTNNB1 and LRP1B in the IRGPI-high group was found to be significantly higher than that in the IRGPI-low group. Previous study indicated that TP53 or LRP1B mutations are associated with higher TMB and worse survival in HCC [51]. CTNNB1 mutation-associated aldolase A (ALDOA) phosphorylation promotes HCC cell proliferation, which can be mutually verified with the results of our survival analysis [52].

Conventional biomarkers for predicting the efficacy of immunotherapy such as PD-L1 expression, TMB, and MSI usually focus on the expression of tumor cell immunosuppressive molecules and the formation of neoantigens. The effectiveness of immunotherapy needs the support from the immune system, which leads to insufficient accuracy of traditional biomarkers. In HCC, tumoral PD-L1 expression was not predictive for response to nivolumab or pembrolizumab [53, 54]. HCC is usually accompanied by low TMB, and TMB as a biomarker to predict response to immunotherapy in HCC is not supported by available data [55]. Similarly, the prevalence of MSI-high status is rare in HCC [56]. Notably, high TMB is not necessary associated with better response to ICIs. One reason is that a large number of passenger mutations in the genome cannot produce tumor-specific antigen peptides that can be recognized by the immune system. A study found that of the 75,179 unique neoantigens were identified in tumor cells, only 28 (0.04%) occurred in patients who were benefited from ICIs [57].These findings indicated that most neoantigens correlated with immunotherapy benefit are patient specific. In addition, neoantigens needs to be processed and presented by antigen presenting cells (APC) to effectively activate CD8^+^ T cells, which indicates that the number and function of infiltrating immune cells in tumor microenvironment are more important for the effectiveness of immunotherapy [58]. As a result, the efficacy prediction of immunotherapy must be considered from multiple perspectives. Whether there are sufficient quantity and quality of immune cells in the tumor microenvironment to produce a killing effect on the tumor is more important. Herein, we used R packages “CIBERSORT” and “ESTIMATE” to systematically analyze the immune infiltrating cells and immune function in the tumor microenvironment. In addition, we also evaluated the expression of immune checkpoint molecules between different groups. The results showed that two groups had different immune landscapes. The immune microenvironment in the IRGPI-low group has sufficient immune cell infiltration and is inflammation-promoting, which is conducive to the progress of the anti-tumor immune response. According to a synergistic analysis with immunosuppressive molecules, tumor cells of IRGPI-low group typically exhibit a high level of PD-L1. This implies that immune cells in the tumor microenvironment are functionally suppressed, and ICIs can effectively awaken immune effector cells to kill tumors.

In the IRGPI-high group, the abundance of immune infiltrating cells in the tumor microenvironment is low and the expression of CTLA-4 is increased. CTLA-4 is an inhibitory receptor constitutively expressed by regulatory T cells (Tregs) that suppresses effector T cell proliferation, activation and migration [59]. Due to the structural similarity, CTLA-4 can competitively bind ligand with CD28, thus inhibiting the transmission of T cell activation signal. In addition, CTLA-4 can also interfere with signal transduction of TCR/CD3 through dephosphorylation [60]. Blockage of activation signal transduction leads to the reduction of T cell proliferation and cytokine secretion, which leads to T cell inactivation and inability to maintain anti-tumor activity. [61]. CTLA-4 can also reduce the adhesion ability of T cells by down-regulating the expression of adhesionmolecules, making T cells more difficult to infiltrate into the tumor microenvironment [62]. IRGPI-high patients are usually accompanied by higher frequency of CTNNB1 mutation. CTNNB1 mutation can significantly reduce the number of activated immune cells and secretion of immune-stimulating molecules in HCC. In contrast, the number of M2-type macrophages and active immunological depletion pathways increased significantly with CTNNB1-mutant [63]. These findings may help explain why IRGPI-high patients have higher immune exclusion scores and are insensitive to immunotherapy. Instead of blindly employing ICIs in IRGPI-high patients, increasing the number of immune infiltrating cells in the tumor microenvironment should be emphasized. Therefore, patients in the IRGPI-high group may require pretreatment with other treatments to transform cold tumors into hot tumors before using ICIs. Radiotherapy and anti-angiogenesis therapy can increase the number of immune cells by destroying the immune barrier and reshaping the tumor microenvironment [64]. The complementary effects of the two immunological up-regulated pathways (CTLA-4 and PD-1/PD-L1) may result in a synergistic impact when different ICIs are combined. The multi-ICIs or the combination of ICIs and other therapies has become a novel strategy to treat HCC [65]. The IRGPI-high group has high expression of CTLA-4 and a small number of CD8 + T cells in the tumor microenvironment, which may be an indication for a combination of anti-PD-1/PD-L1 and anti-CTLA-4. Currently, several clinical trials combining PD-1 and CTLA-4 inhibitors have shown promising results, suggesting a novel strategy for overcoming the ineffectiveness of single ICIs [66, 67].

There are certain limitations of this study. Because there is no dataset on immunotherapy for HCC, the model to predict response to immunotherapy can only be verified by other types of tumors. This method is mainly based on the special pharmacological effect of immunotherapy. The mechanism of immunotherapy is to restore the anti-tumor activity of the immune system suppressed by immune checkpoint. The anti-tumor activity is non-specific, which is why immunotherapy have a broad-spectrum therapeutic effect. Although the tumor type and microenvironment are not the same, atezolizumab has been approved for the treatment of urothelial carcinoma and HCC. The finding of the investigation demands clinical trial-based verification in a larger HCC cohort receiving immunotherapy.

In summary, we comprehensively analyzed the immune-related genes of HCC, providing a clear picture of immune landscape in HCC. The difference in immune-related gene patterns was found to be correlated to tumor heterogeneity and microenvironment complexity. Thus, our systematic analysis of immune-related gene pattern has crucial clinical implications. In addition, IRGPI can facilitate the identification of potential candidates for immunotherapy.

## Conclusion

In this study, we established a prognostic model based on immune-related genes. This model can predict the prognosis of patients with HCC, and more importantly, can examine the response to ICIs. In addition, we also analyzed the influence of IRGPI on immune infiltrating cells in the tumor microenvironment and the association with other biomarkers. Hence, IRGPI can be as a potential biomarker to help clinicians better identify HCC patients who could benefit from immunotherapy.

## Data Availability

The datasets generated during and analyzed during the current study are available in the TCGA and GEO repository, https://portal.gdc.cancer.gov/, http://research-pub.gene.com/IMvigor210CoreBiologies, https://www.ncbi.nlm.nih.gov/geo/query/acc.cgi?acc=GSM1648114, https://www.ncbi.nlm.nih.gov/geo/query/acc.cgi?acc=GSE78220.

## Abbreviations

ICIs: Immune checkpoint inhibitors
HCC: Hepatocellular carcinoma
IRGPI: Immune-related gene-based prognostic index
TCGA: The Cancer Genome Atlas
GEO: Gene Expression Omnibus
WGCNA: Weight Gene Co-expression Network Analysis
TIDE: Immune Dysfunction and Exclusion
TMB: Tumor mutation burden
MSI: Microsatellite instability
TIL: Tumor infiltrating lymphocytes
IRGs: Immune-related genes
ImmPort: Immunology Database and Analysis Portal
DEIRGs: Differentially expressed immune-related genes
GO: Gene Ontology
KEGG: Kyoto Encyclopedia of Genes and Genomes
FDR: False discovery rate
ME: Module eigengene
MM: Module membership
OS: Overall survival
LASSO: Least Absolute Shrinkage and Selection Operator
CC: Cellular component
MF: Molecular function
BP: Biological process
MAPK: Mitogen-activated protein kinase
IFN: Interferon
ALDOA: Aldolase A
PD-L1: Programmed cell death-ligand 1
CTLA-4: Cytotoxic T-lymphocyte-associated protein 4
CR: Complete response
PR: Partial response
SD: Stable disease
PD: Progressive disease
ORR: Objective response rate
